# An octavalent vaccine provides pregnant gilts protection against a highly virulent porcine parvovirus strain

**DOI:** 10.1186/s12917-020-2272-3

**Published:** 2020-02-12

**Authors:** Erwin van den Born, Paul P. M. van den Elzen, Emma van Kilsdonk, Mathieu J. H. Hoeijmakers, Ruud P. A. M. Segers

**Affiliations:** MSD Animal Health, P.O. Box 31, 5830 AA Boxmeer, The Netherlands

**Keywords:** Porcine parvovirus, Vaccination, Pregnant gilts, Mummification

## Abstract

**Background:**

Porcilis® Ery+Parvo+Lepto is an octavalent inactivated ready-to-use vaccine that contains *Erysipelothrix rhusiopathiae* (Ery), porcine parvovirus (PPV), and six serogroups of *Leptospira* (Lepto). The efficacy of Porcilis® Ery + Parvo+Lepto against reproductive problems associated with porcine parvovirus (PPV) infection was evaluated in pregnant gilts. For this, a group of ninegilts was vaccinated twice (at 5 and 6 months old) with Porcilis® Ery + Parvo+Lepto (Group 1), while a group of eight gilts was included as unvaccinated controls (Group 2). All pigs were artificially inseminated 4 weeks after the second vaccination. They were challenged during early gestation with PPV-27a, a virulent cluster D strain, and euthanized to collect their offspring by hysterectomy around day 90 in pregnancy. Antibody responses against PPV in gilts were measured, and the presence of PPV in progeny was also determined.

**Results:**

No clinical signs were observed after vaccination. After PPV challenge, all foetuses from the vaccinated gilts were alive (132/132), while in the unvaccinated group only 41% were alive (46/112), 19.6% were dead and 39.4% of the offspring (44/112) were mummified. PPV could be detected by qPCR in 14% of the progeny from vaccinated gilts at an average of 4.7 log_10_/ml, whereas this was significantly higher in the control group, where 90% of the progeny were PPV positive, with titres of 9.8 log_10_/ml on average.

**Conclusions:**

The present study demonstrates that vaccination of gilts with Porcilis® Ery + Parvo+Lepto was safe and induced an immune response sufficient to protect progeny against PPV by reducing transplacental infection.

## Background

Porcine parvovirus (PPV) is present in the majority of pig herds worldwide and is the most common and important cause of infectious infertility. It causes reproductive losses represented by stillbirth, mummification, embryonic death, infertility (SMEDI-syndrome) and delayed return to oestrus. Parvovirus infection in nonpregnant adult pigs rarely causes clinical signs, but during pregnancy the virus can cross the placental barrier to infect the rapidly dividing tissues of the embryos and foetuses. The outcome of the PPV infection of the foetus varies with the progression of gestation [1]. Foetuses infected early in pregnancy usually die, resulting in their mummification or resorption, whereas foetuses infected at a later timepoint develop antibodies against PPV and may survive, but as a result of the infection may be weak at birth [2].

PPV is a member of the genus *Parvovirus* within the family *Parvoviridae*. It is a small, nonenveloped, single-stranded DNA virus. Although PPV replicates by using the host DNA replication machinery, it exhibits a relatively high mutation rate that is more similar to RNA viruses [3]. PPV variants are continuously evolving and this raises the question whether established vaccine strains that were isolated several decades ago are still providing protection [4]. Such a more divergent variant, PPV-27a, was isolated in Germany in 2001 and has now been phylogenetically categorized as a cluster D strain [5, 6]. A high rate of foetal mummification was observed after infection of pregnant gilts with strain PPV-27a [4].

Swine erysipelas (caused by *Erysipelothrix rhusiopathiae*) is a disease of greatest prevalence and economic importance [7]. Leptospirosis (caused by *Leptospira interrogans* sensu *lato*) is also a cause of reproductive failure in pigs worldwide [8]. Vaccines against erysipelas and parvovirus are routinely used in the pig industry whereas leptospirosis vaccines are used less commonly. For user convenience and to reduce the number of injections given to gilts and sows, a ready-to-use combination product was developed by adding six relevant swine Leptospira antigens (serogroups Canicola, Icterohaemorrhagiae, Australis (Bratislava), Grippotyphosa, Pomona and Tarassovi) to an existing vaccine, Porcilis® Ery + Parvo. This new octavalent inactivated vaccine, Porcilis® Ery + Parvo+Lepto, was found to be safe and efficacious against each of the infectious agents [9, 10]. Naïve animals require two vaccinations of this vaccine, 4 weeks apart. Immunity can be maintained by revaccinating pigs according to the schedule described in the product leaflet of Porcilis® Ery + Parvo+Lepto.

The goal of this study was to evaluate the efficacy of Porcilis® Ery + Parvo+Lepto, containing the PPV-014 strain, against reproductive disorders associated with PPV infection of pregnant gilts that were challenged during early gestation with the contemporary and virulent PPV-27a strain.

## Results

### Clinical signs and serological response after vaccination with Porcilis® Ery + Parvo+Lepto

No clinical signs were observed after vaccination of the gilts with Porcilis® Ery + Parvo+Lepto.

**Fig. 1:**
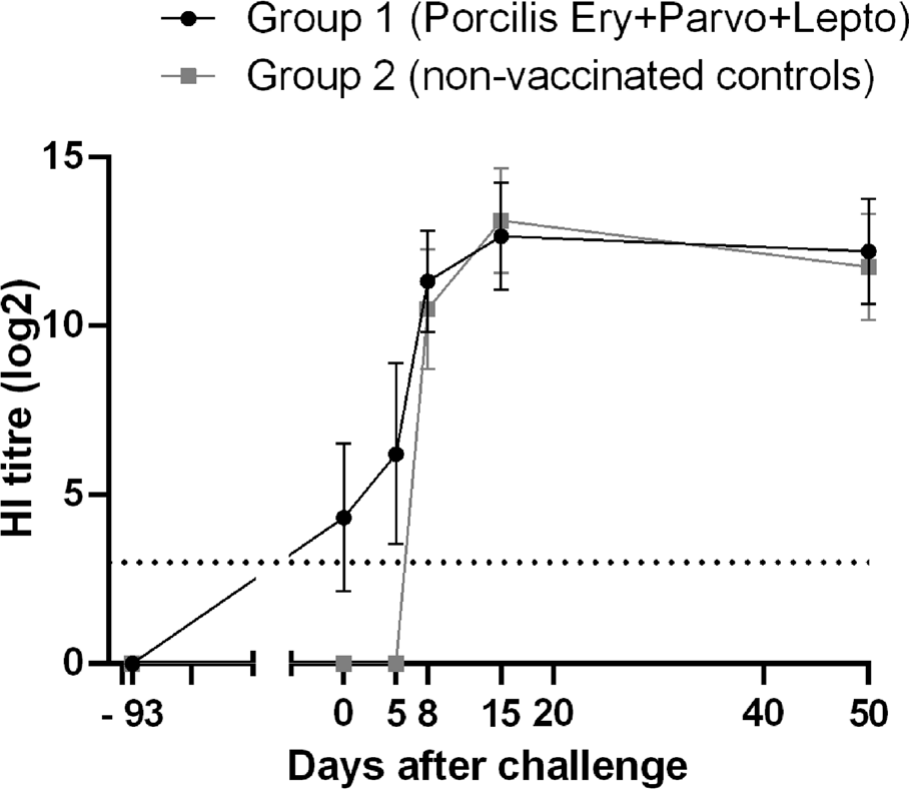
HI antibody response in gilts after vaccination and PPV challenge. Error bars represent the standard deviation. Dotted line is the detection limit (values < 3 log_2_)

At the first vaccination, all gilts were negative for PPV antibodies. At the time of challenge, 93 days after the first vaccination, one-third of the gilts vaccinated with Porcilis® Ery + Parvo+Lepto had developed detectable HI antibodies, while none of the challenge control gilts in Group 2 showed a measurable HI antibody response (Fig. 1). After challenge, all gilts developed HI titres against PPV.

### Vaccination of gilts with Porcilis® Ery + Parvo+Lepto protected the progeny from PPV-induced death

**Fig. 2:**
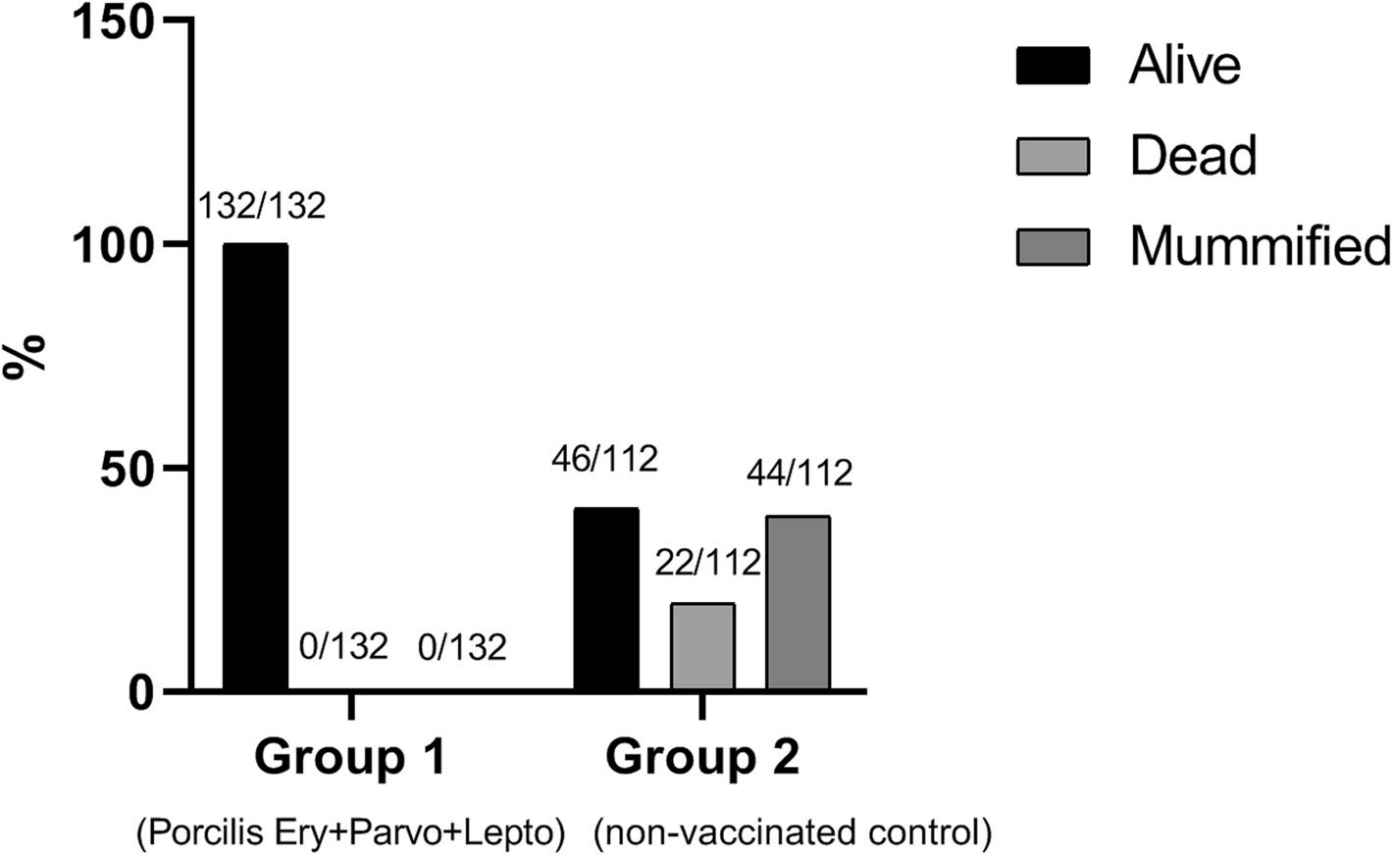
Vitality of the progeny at around day 90 in pregnancy

Vitality of the progeny is showed in Table 1. A total of 132 foetuses were collected from the pregnant gilts in Group 1 of which all were alive. The control gilts had a total of 112 foetuses of which 46 were alive, 22 dead and 44 mummified. The calculated percentages of vital foetuses per group clearly demonstrate that a reduction in foetal mortality can be achieved by vaccinating gilts with Porcilis® Ery + Parvo+Lepto (Fig. 2).

**Table 1:** Vitality and presence of PPV in foetuses per sow.

Group	Sow No.	Vitality in progeny				PPV infection in progeny				
						PPV Ag Pos (HA titre)	PPV Ab Pos (HI titre)	PPV DNA Pos (qPCR)	PPV-Pos HI,HA, and/or qPCR	
		Total	Alive	Dead	Mummified					% Protected
1	226	11	11	0	0	0	0	0	0	
	227	15	15	0	0	0	0	2	2	
Porcilis Ery + Parvo + Lepto	229	8	8	0	0	0	0	0	0	
	230	21	21	0	0	0	0	4	4	
	231	14	14	0	0	0	0	2	2	
	232	17	17	0	0	0	0	2	2	
	233	13	13	0	0	0	0	3	3	
	235	16	16	0	0	0	0	4	4	
	236	17	17	0	0	0	0	2	2	
	Total	132	132	0	0	0	0	19	19	
	Percentage	100%	100%	0%	0%	0%	0%	14%	14%	85.6%
2	2	16	3	2	11	11	4	15	16	
	3	12	4	4	4	4	4	11	12	
Control	4	16	1	9	6	6	3	16	16	
Non vac.	5	18	12	3	3	3	5	18	18	
	6	10	9	1	0	0	0	4	4	
	9	17	8	3	6	6	0	12	12	
	10	13	0	0	13	13	0	13	13	
	11	10	9	0	1	1	0	8	8	
	Total	112	46	22	44	44	16	97	99	
	Percentage	100%	41%	20%	39%	40%	15%	90%	90%	10%

*Ag* antigen; *Ab* antibody; *Pos* positive

### Vaccination of gilts with Porcilis® Ery + Parvo+Lepto reduced transplacental infection

The infectivity data is summarized in Table 1. It was established that 90% of the offspring of the control gilts were infected by PPV, as material taken from these foetuses was PCR, HA, and/or HI positive. In contrast, 14% of the offspring of the Porcilis® Ery + Parvo+Lepto vaccinated gilts were PPV positive by PCR, and no response was detectable by HA or HI assays.

When comparing the PPV DNA load in the foetuses of the group 1 and group 2 gilts, it was observed that vaccination with Porcilis® Ery + Parvo+Lepto reduced the average viral DNA content in PPV-positive foetal tissue homogenates significantly, from 9.8 log_10_/ml to 4.7 log_10_/ml (*P* < 0.0001).

## Discussion

Vaccination ensures active immunity against PPV in gilts and sows and is the most effective tool to prevent PPV-induced losses in the breeding herd. This study demonstrates that the octavalent Porcilis® Ery + Parvo+Lepto vaccine is safe, as no clinical signs were observed after vaccination in any of the gilts. The study also demonstrates that the vaccine protects against reproductive losses associated with PPV infection of pregnant gilts. The gilts were challenged with PPV-27a, a more contemporary cluster D strain, while the PPV strain in the vaccine belongs to cluster A [1]. The VP1 protein of PPV-27a shows a 97.8% sequence identity compared to PPV-014 (results not shown). This observation is in accordance with previously reported studies, which demonstrate that the German field isolates such as PPV-27a differ genetically from various vaccine strains [3, 5]. Even though our vaccine strain is not closely related to the challenge strain used in the study, Porcilis® Ery + Parvo+Lepto conferred good protection after experimental infection. Animals were fully protected from clinical disease, however PPV DNA was detected in foetuses from vaccinated gilts, albeit at a much lower level than in the unvaccinated controls. This observation is in accordance with previous studies that described viral shedding in vaccinated, clinically protected animals following heterologous or homologous PPV challenge [11, 12].

Previous reports have described detection of PPV-specific antibodies as early as day 6 post-infection and the peak antibody titres on days 14–21 after infection [1], which is in agreement with our findings (Fig. 1). Foerster et al. demonstrated that vaccination with inactivated PPV-27a prevented foetal death after homologous virus challenge with PPV-27a. However, a substantial increase in antibody titres was observed after infection, indicating virus replication in the immunised animals [12]. In our study, both groups responded serologically after challenge, reaching similar peak levels of HI antibodies.

PPV neutralization assays would have been useful to confirm that the detected HI antibodies were neutralizing antibodies. Virus isolation from mummified piglets would also have been useful to verify that PPV detected by PCR was infectious or not. Unfortunately, these techniques could not be performed for this study.

Despite the virulence described for strain PPV-27a after experimental infection of pregnant gilts [4], the mortality rate among the foetuses of the vaccinated and unvaccinated gilts differed significantly: in Porcilis® Ery + Parvo+Lepto vaccinated gilts (group 1) 100% of the foetuses were alive and unaffected, while in unvaccinated gilts (group 2) more than half (58.9%; 66 out of 112) of the foetuses were dead or mummified (Fig. 2). These results demonstrate the effectiveness of the two-dose vaccination strategy for Porcilis® Ery + Parvo+Lepto.

Regarding PPV infection of the progeny, more foetuses in the control group were infected by the PPV-27a strain than the foetuses in the Porcilis® Ery + Parvo+Lepto vaccinated group (90% versus 14% respectively; Table 1) and also more severely (i.e. higher level of viral copies in group 2). This result agrees with previous studies, which also used the virulent PPV-27a strain and found high foetus mortality [4, 11, 12], although it has to be noted that positive PCR results may represent the transplacental transfer of non-infectious virus or PPV DNA [4].

Porcilis® Parvo and Porcilis® Ery+Parvo, two other PPV-containing vaccines, carry the same cluster A parvovirus strain as Porcilis® Ery + Parvo+Lepto. It is therefore anticipated that these vaccines will provide protection against more recent and virulent cluster D strains like PPV-27a, similar to Porcilis® Ery + Parvo+Lepto. An alternative study in which pregnant gilts were vaccinated once with Porcilis® Ery + Parvo before insemination, and subsequently challenged with PPV-27a has confirmed this (unpublished). This is a relevant observation, because in recent years there is a predominance of the European strains in related clusters C and D [3].

## Conclusions

In summary, vaccination of gilts with Porcilis® Ery + Parvo+Lepto was safe and effectively reduced foetal mortality caused by infection of pregnant pigs by the virulent PPV-27a strain.

## Methods

### Experimental design of the animal trial

Two groups of randomly selected PPV-negative healthy gilts (Fokbedrijf VOF Jacobs, The Netherlands) were included in the study (Table 2). After an acclimatization period, animals in Group 1 (*n* = 9) were vaccinated intramuscularly in the neck with Porcilis® Ery + Parvo+Lepto (batch IP141910) when they were approximately 5 months old and were revaccinated 4 weeks later. The gilts in Group 2 (*n* = 8) were not vaccinated and served as challenge controls. In the 3 weeks following the last vaccination, the oestrus of all gilts was synchronized by Regumate treatment following the manufacturer’s instructions (MSD Animal Health). Maprelin was administered 2 days after the last Regumate treatment and 3 days before the first insemination to enhance the success of the insemination. Four weeks after the last vaccination, all pigs were artificially inseminated with PPV-free sperm on two to three consecutive days. Around day 40 in pregnancy, gilts were challenged with the virulent strain PPV-27a. Around day 90 in pregnancy, gilts were exsanguinated after having been anaesthetised by electrocution and the offspring were obtained by hysterectomy. If the foetus was mummified, the entire foetus was collected. Serum was collected from the live foetuses and body fluid from dead foetuses. Serum, body fluid samples and homogenates of the mummified foetuses were tested for the presence of PPV and/or PPV-specific antibodies. Blood samples of the gilts were collected before the first vaccination, at challenge and 5, 8 and 15 days after challenge and at the end of the experiment. Blood samples were tested for the presence of antibodies against PPV. Treatment, housing and husbandry conditions conformed to the guidelines of the European Union for animal welfare and the study was performed according to *Good Experimental Practices*.

**Table 2:** Overview of groups and their treatment.

		Vaccination at 5 months of age		Vaccination at 6 months of age		Challenge infection at 40 days in pregnancy	
Group	No. of gilts	Vaccine	Dose/ Route	Vaccine	Dose/ Route	challenge strain	Dose/ Route
1	9	EPL	2 ml IM	EPL	2 ml IM	27a	2 × 1 ml IN 2 ml IM
2	8	–	–			27a	2 × 1 ml IN 2 ml IM

*EPL* vaccine containing *Erysipelotrix rhusiopathiae*, porcine parvovirus, and Leptospira; *IM* intramuscular; *IN* intranasal

### Vaccine

The Porcilis® Ery + Parvo+Lepto vaccine (MSD Animal Health) contains the Diluvac Forte® adjuvant, inactivated *Erysipelothrix rhusiopathiae*, inactivated porcine parvovirus, and six *Leptospira interrogans* serogroups, i.e. Canicola, Icterohaemorrhagiae, Australis (Bratislava), Grippotyphosa, Pomona and Tarassovi.

### PPV challenge material

PPV 27a strain, kindly provided by Prof. Truyen [4], was cultured on SK6 cells and stored at − 70 °C until use. Shortly before use, the challenge material was diluted using 0.01 M PBS to contain a concentration of 6.0 log_10_ TCID_50_/ml. The challenge consisted of 2 ml of the virus given intranasally and 2 ml intramuscularly. Thus, a calculated total dose of 6.6 log_10_ TCID_50_ per animal.

### Sample processing

Blood was collected in vacutainers without anti-coagulant and allowed to coagulate. Serum was prepared from the clotted blood samples by centrifugation (3000x g, 10 min), aliquoted and stored at − 20 °C until use.

Homogenates of the entrails of the foetus (20% w/v) were individually prepared in 200 mM glycine buffer pH 9.5 using an IKA tube drive station and stored at − 20 °C until use.

### Haemagglutination inhibition (HI) assay for the detection of antibodies to PPV

Sera from gilts and body fluid or sera from foetuses were tested for the presence of antibodies that inhibit the haemagglutination of guinea pig red blood cells by PPV in a HI assay. Two-fold dilution series of 50 μL of the test samples were prepared in micro-titre plates and mixed with an equal volume of a solution containing 4–8 HA units of PPV. A positive and negative control was included. After incubation for 45 min at room temperature, red blood cells were added to a final concentration of 0.33%. After incubation overnight at 2–8 °C, plates were examined for red blood cell agglutination. The titre of antibodies inhibiting agglutination of the red blood cells by PPV was expressed as log_2_. The detection limit was 4 log_2_. Samples below that value were considered negative. To be able to calculate the average of PPV antibody titres, an HI titre of < 4 log_2_ was defined as a titre of 3 log_2_. An HI titre of > 15 log_2_ was defined as a titre of 16 log_2_. For representation purposes, an average titre of 3 log_2_ was set at 0.

### Haemagglutination (HA) assay for the detection of PPV

The amount of PPV in mummified piglet homogenates was quantified using its ability to agglutinate guinea pig red blood cells (HA assay). The HA test was performed on supernatant obtained after centrifugation of the homogenate at 1000x g for 10 min at 4 °C. Two-fold dilution series of 50 μL of test sample in 50 μL of HA buffer (0.1% (v/v) BSA in PBS) were mixed in V-shaped micro titre plates. An equal volume of 50 μL of guinea pig red blood cells was added. A positive and negative control was included. After incubation for 2 h at 2–8 °C plates were examined for agglutination of the red blood cells. The antigen content was expressed as log_2_ HA unit per 50 μL. The detection limit was 1 log_2_. Samples showing < 1 log_2_ were considered negative.

### PPV-specific quantitative polymerase chain reaction (qPCR)

Sera or body fluid from progeny or mummy-homogenates were analyzed by qPCR for the presence of PPV DNA. Amplified DNA-fragments were detected by using SYBR green that only fluoresces when bound to DNA. The amount of PPV DNA in a sample was determined by including in each test a 10-fold serial dilution of a reference standard (plasmid pPPV-02 containing a 4.5 kb *Pst*I-*Dra*I fragment of PPV strain NADL-8). A melt curve was used to analyze the specificity of the signal. Samples giving a C_q_ value below 40 and a specific melt curve were considered positive. Samples not fulfilling both criteria were considered negative. The PPV content of positive samples was expressed as log_10_ copies per ml.

### Statistical analysis

Viral load data in foetuses were analysed with a mixed model ANOVA with group as fixed effect and accounting for the correlation within a litter by including gilt, representing the litter, as random effect. The Dunnett’s multiple comparison method was used in comparing each vaccine group to the control. In the calculations qPCR results <LOQ where replaced by LOQ-0.3 (log_10_), reflecting one duplication in the amplification. This is considered as a conservative approach in estimating the difference in viral load between the vaccine and control group since the majority in the vaccine group was <LOQ and in the control almost all were > LOQ.

Tests were 2-sided at a significance level of 5%. SAS version 9.3 (SAS Institute Inc., Cary, NC, USA) was used for the statistical analysis.

## Data Availability

The datasets generated and/or analysed during the current study are not publicly available due to the size of the dataset (244 piglets and 17 gilts), but are available from the corresponding author on reasonable request.

## Abbreviations

ANOVA: Analysis of variance
BSA: Bovine serum albumin
DNA: Deoxyribonucleic acid
HA: Haemagglutination
HI: Haemagglutination Inhibition
LOQ: Limit of quantification
PBS: Phosphate-buffered saline
PPV: Porcine parvovirus
SK6 cells: Swine Kidney 6 cells
TCID_50_: Tissue culture infective dose (50%)
